# Supplementary material for: Distinct Roles of Estrogen Receptors in the Regulation of Vitellogenin Expression in Orange-Spotted Grouper (Epinephelus coioides)
Source: Int J Mol Sci. 2022 Aug 3;23(15):8632. doi: 10.3390/ijms23158632 (PMC9369318; doi:10.3390/ijms23158632)
Supplement: Supplementary file 1 [file ijms-23-08632-s001.zip › Supplementary figures S1.pdf]

Supplementary figure S1:

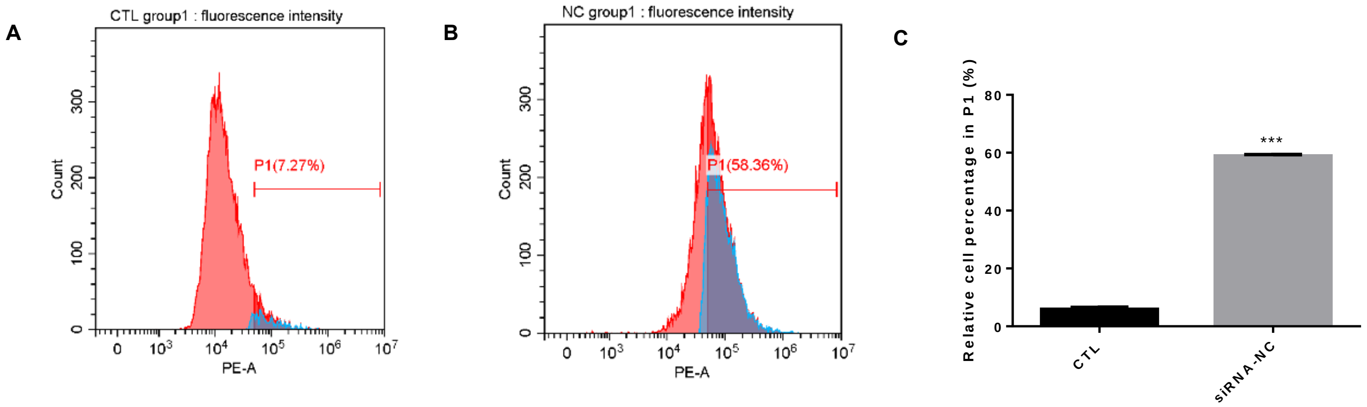

Figure S1. Flow-cytometry histograms showing siRNA transfection result of primary hepatocytes culture of orange-spotted grouper. (A) Fluorescence intensity graph of the control group. (B) Fluorescence intensity graph of the siRNA-NC-Cy3 group. (C) Compared with the control group, the number of cells in the strong fluorescence range (P1) in the siRNA-NC-Cy3 group increased significantly. The ordinate represents the number of cells, the horizontal coordinate represents the relative intensity of PE-A fluorescence signal, and P1 represents the relative percentage of the number of cells within the strong fluorescence signal range. Data are expressed as the mean ± SEM of triplicate experiments. Asterisks (*) indicate statistical differences (*$P < 0.05$, **$P < 0.01$, ***$P < 0.001$)
