# Supplementary material for: Distinct Roles of Estrogen Receptors in the Regulation of Vitellogenin Expression in Orange-Spotted Grouper (Epinephelus coioides)
Source: Int J Mol Sci. 2022 Aug 3;23(15):8632. doi: 10.3390/ijms23158632 (PMC9369318; doi:10.3390/ijms23158632)
Supplement: Supplementary file 1 [file ijms-23-08632-s001.zip › Supplementary figures S2.pdf]

Supplementary figure S2:

```
GAGCTCCTGTTGTAACTGGTTTTATTTTACACTTTATTTTACCACAAGTGTTGGAAAATAATCCTTACAC -1364
TTACAGTTTATTAGTTTTCCTGGTAGTGTTTCAGCTCAAGGTTTTTTGTTTTTTTTTACCTTGATGCCAT -1293
GTCCCCAAACATACAAGGACTCATGGGATCCCTGACAATCACAGACCTGGAAATTTTGTGCAGTAAACAT -1222
                 AP-1

GAACATTGATTTCCTAAGCCTTTATTTGCATAGATACTCTGCCCCTTTGCTTTGATTGGTTCATATATAA -1151
TGTTCTGTTTAATCTCCGGCTTGACCTGATAAAGTAATGATTGACTGGTCCATACTGTTATTCTGTAATA -1080
                    1/2ERE AP-1           AP-1

TATTTTTCAATGAAATGTCATGAGGAAAATGTAAAGAAAAACGTTCCTGTATTTTTTTGTAGTTTAAAAA -1009
AAAAACTTAAGTTCAAGCAGCATCTGATTAAAATAGCCACCGATCGCCCTCATTTGGGATTGATTGTGTA -938
                                            SP-1

ATTATTGGCTGTTGGAATGAGATTTGACCTCCTGCTTTGTTTGCTGTGTTATGGTGACCTGGCAGTAAAA -867
                      1/2ERE#1                       1/2ERE#2

CACACTGCTGTTTGGTTATCGACTTGGCCGCATCTTTAATTTGTTTATCCTCTTTTTTAACCTTTCCACC -796

GTTCCCATTGTATCCAATGAGCCAGGCTAGATAGCTGAGAGTCAGAGAGAGGAGAGAGGCAAGCAGAGAC -725
                     AP-1              AP-1

AGAGGAGACTCAAACAGTGCCAGTGTTGCACGGCAGATGAGAGGATGATTCATGTGTGAGAGGTGAGTAA -654
      AP-1                                     AP-1             AP-1

TGTATCATGGTGGATTTCTGCCTTTTTTTAATAGCCACTGTTGTTTAAAACTATTGTCCTAATGTTTCTC -583

ATTTCCCGAGTGATTTGATTTACTGCAGAGTCTCAGGTGTATCCAATAATGTGGCTACAAATAGACTGCT -512
CATACGTCATGCTGGTAGCATGCTGAGTATCAGATAAATTAAACAGCTTACTGCTAGTTTACAGATGTTT -441
  CREB

TAATCTGTGCACCAGCTGCAACTGGGAAGAGCTTTTCAAAATTAAGAAAACAGGTGAAATAAGAAGAGAG -370
CATTTTATAGGGATGTGAGAGAGAGAGAGAGAGAGAGACAGGACAGGGAAGTTATTTGGGAATCACTA -299
TTTCTCTGTCCATCTTCCTCTCCCCCCTATAGAGTAGATGTATCAGAGGAAAAGAGGGAAACGCTCTGCT -228
GATTTAAAAAAAAAAAAAAAAACTGAAGGAGATTGGGAGGAGTCTTTGTTTTTTGGAGCTTGGTGGGTGG -157
AAGCTCAAGGCAGATAGGGCAAAGGGACTGGAAGAATAGTGGACCCTTCAAGTTGCCTGACAGGCTTTAT -86
CGGTAAAAAGGCAAGAGAGACAGAGAGAGGGAGAGCAGCACTCCCCCCCCCCCAGCAAATGGGAGAGCAA -15
GTCTAAATCATGTTGCATATCAGTGCTGTTGTGCTGTGATGTTGCTCAGGCAGAGCCCGGCGCAGAGCAG +56
              TSS
```

Figure S2. Sequence analysis of the promoter region (1434 bp upstream of TSS) of grouper *era*. The putative binding sites for transcription factors were labeled, in which half EREs were underlined by full lines, whereas others were underlined by dotted lines. The numbers on the right side of each lines indicate the distance from TSS to the rightmost nucleotide of this line. The transcription start site is noted by an arrow and marked as TSS. Half-EREs marked as ERE#1, or ERE#2 indicate the sites that were applied in the site-directed mutagenesis test.
